# Synergy between Human Peptide LL-37 and Polymyxin B against Planktonic and Biofilm Cells of *Escherichia coli* and *Pseudomonas aeruginosa*

**DOI:** 10.3390/antibiotics12020389

**Published:** 2023-02-15

**Authors:** Kylen E. Ridyard, Mariam Elsawy, Destina Mattrasingh, Darien Klein, Janine Strehmel, Carole Beaulieu, Alex Wong, Joerg Overhage

**Affiliations:** 1 Department of Health Sciences, Carleton University, Ottawa, ON K1S 5B6, Canada; 2 Department of Biology, Carleton University, Ottawa, ON K1S 5B6, Canada; 3 Institute of Functional Interfaces, Karlsruhe Institute of Technology, 76344 Karlsruhe, Germany

**Keywords:** LL-37, polymyxin B, antimicrobial resistance, synergy, anti-biofilm, *Pseudomonas aeruginosa*, *Escherichia coli*

## Abstract

The rise in antimicrobial resistant bacteria is limiting the number of effective treatments for bacterial infections. *Escherichia coli* and *Pseudomonas aeruginosa* are two of the pathogens with the highest prevalence of resistance, and with the greatest need for new antimicrobial agents. Combinations of antimicrobial peptides (AMPs) and antibiotics that display synergistic effects have been shown to be an effective strategy in the development of novel therapeutic agents. In this study, we investigated the synergy between the AMP LL-37 and various classes of antibiotics against *E. coli* and *P. aeruginosa* strains. Of the six antibiotics tested (ampicillin, tetracycline, ciprofloxacin, gentamicin, aztreonam, and polymyxin B (PMB)), LL-37 displayed the strongest synergy against *E. coli* MG1655 and *P. aeruginosa* PAO1 laboratory strains when combined with PMB. Given the strong synergy, the PMB + LL-37 combination was chosen for further examination where it demonstrated synergy against multidrug-resistant and clinical *E. coli* isolates. Synergy of PMB + LL-37 towards clinical isolates of *P. aeruginosa* varied and showed synergistic, additive, or indifferent effects. The PMB + LL-37 combination treatment showed significant prevention of biofilm formation as well as eradication of pre-grown *E. coli* and *P. aeruginosa* biofilms. Using the *Galleria mellonella* wax worm model, we showed that the PMB + LL-37 combination treatment retained its antibacterial capacities in vivo. Flow analyses were performed to characterize the mode of action. The results of the present study provide proof of principle for the synergistic response between LL-37 and PMB and give novel insights into a promising new antimicrobial combination against gram-negative planktonic and biofilm cells.

## 1. Introduction

Antibiotic resistance is one of the biggest public health threats worldwide, as announced by the World Health Organization (WHO). The rise in resistance, including multidrug-resistant and pan-resistant bacteria, is limiting the success of many currently available treatment options for bacterial infections. Accordingly, the research and development of new antibiotics and therapeutic options are crucial [1]. In 2017, the WHO drew attention to twelve families of bacteria for which we urgently require new antibiotics as treatments. These bacteria largely comprise gram-negative bacteria, including carbapenem-resistant *Pseudomonas aeruginosa* and *Enterobacteriaceae*, as well as ESBL-producing *Enterobacteriaceae* [2]. In accordance with the WHO recommendations, this study focused on *E. coli* and *P. aeruginosa* as targets of combination therapy. 

*E. coli* and *P. aeruginosa* are both gram-negative, opportunistic pathogens that are responsible for hospital-associated infections [3]. *E. coli* is a commensal bacterium found in the gut of vertebrates, although as an opportunistic pathogen, it does cause both intestinal and extra-intestinal infections including hemolytic uremic syndrome (HUS), urinary tract infections (UTIs), and bacteremia [4]. *P. aeruginosa* also poses major challenges to patients, particularly those with cystic fibrosis, burn and chronic wounds, and chronic obstructive pulmonary disorder [3]. Both bacteria display several resistance mechanisms, such as antibiotic inactivating enzymes [5] and antimicrobial resistance plasmids [6,7], that encourage the evasion of antimicrobial agents and the emergence of multidrug-resistant pathogens. In 2019, *E. coli* and *P. aeruginosa* were two of the six pathogens associated with approximately 3.57 million of the estimated 4.95 million global deaths related to antimicrobial resistance [8]. 

Both *E. coli* and *P. aeruginosa* are capable of producing biofilms, microbial aggregates that can adhere to biotic or abiotic surfaces [9,10]. Biofilms are commonly associated with wound infections [3], intestinal infections, and medical device-associated infections, including those associated with ventilator tubes, catheters, and prosthetic equipment [3,9]. Due to their distinct morphology, physiology, and gene expression, biofilms display different degrees of antimicrobial resistance in comparison with planktonic cells of the same bacterium [11]. In fact, bacteria in a biofilm state can be up to 1000 times more resistant than planktonic bacteria of the same species [11]. There are several proposed mechanisms driving the increased resistance of biofilms to antimicrobial agents, including reductions in the penetration and diffusion of drugs within biofilm structures, as well as the creation of an immune privileged environment that facilitates the growth of persister cells [3]. The increased resistance in this state has allowed biofilm cells to survive even in the presence of antibiotic therapy [10]. Therefore, ideal novel therapeutic agents would have anti-biofilm effects in addition to the killing of planktonic cells. 

One proposed method to combat the rise in bacterial resistance is to combine antimicrobial agents that exhibit synergistic effects. Likewise, combination therapy has been suggested to be a promising method to treat biofilm-associated infections [11]. AMPs have shown success in combination with conventional antibiotics [12,13,14]. In fact, it has been suggested that the in vivo success of an antimicrobial agent may be partly due to the synergistic effects it experiences with AMPs of the host’s immune defenses [12]. This study looked specifically at the AMP, LL-37, and its combination with different classes of antibiotic. 

LL-37 is an effector of the human innate immune response that exhibits moderate antimicrobial activity [15], high anti-biofilm properties [16], as well as immunomodulatory effects [17]. LL-37 uses membrane disruption as its primary mode of action against planktonic cells. The net positive charge of +6 allows for LL-37 to bind to the anionic membranes of bacteria. Once bound, LL-37 disrupts the integrity of the bacterial membranes leading to cell lysis and subsequent cell death [15]. To combat biofilms, LL-37 is proposed to employ several mechanisms, including interference of the initial attachment of cells, inducing cell motility, and disruption of quorum-sensing molecules within the biofilm structure [16]. 

LL-37 has been previously reported to display synergy with antibiotics against resistant *P. aeruginosa* and *E. coli* [18], however, there is limited information pertaining to the synergistic effects of antibiotic + LL-37 combinations against biofilm cells. In this study, we first investigated the synergistic effects between LL-37 and several antibiotics against lab strains of *E. coli* and *P. aeruginosa*. PMB, as the antibiotic that displayed the strongest synergy in combination with LL-37 in the preliminary tests, was chosen for further examination. Herein, we tested the synergistic effects of the PMB + LL-37 combination against drug-resistant and clinical isolates (CIs) of *E. coli* and *P. aeruginosa*. In addition, we examined the synergy against biofilm cells, including the capacity to prevent and eradicate them. Finally, we observed the synergistic effect of LL-37 and PMB using an in vivo model and used flow cytometry to characterize the mode of action.

## 2. Results

### 2.1. Synergy Testing of Different Classes of Antibiotic with LL-37 against E. coli MG1655 and P. aeruginosa PAO1

Initial synergy studies were performed to identify antibiotics that displayed synergy with LL-37 against *E. coli* MG1655 and *P. aeruginosa* PAO1 laboratory strains. The minimal inhibitory concentrations (MICs) (Table 1) were used to calculate the fractional inhibitory concentration indices (FICI) which are presented in Table 2. Polymyxin B (PMB) exhibited the strongest synergistic activity with LL-37 (FICI ≤ 0.5). Of the antibiotics tested, PMB was the sole antibiotic that displayed a synergistic response against both *E. coli* MG1655 and *P. aeruginosa* PAO1. Ciprofloxacin, although demonstrating moderate synergy against *E. coli* MG1655, had only an additive response against *P. aeruginosa* PAO1 (0.5 < FICI ≤ 1.0). Non-synergistic activities were reported for ampicillin, tetracycline, gentamicin, and aztreonam against both bacterial strains. All non-synergistic responses were classified as additive, except for the combinations, gentamicin + LL-37 and aztreonam + LL-37 against *P. aeruginosa* PAO1, which were indifferent (1.0 < FICI ≤ 4.0). Based on the results from the initial synergy testing, the follow-up experiments focused on investigating the synergistic effect of PMB and LL-37 against *E. coli* and *P. aeruginosa* bacterial strains, with a strong emphasis on the anti-biofilm capabilities.

### 2.2. Testing of PMB + LL-37 Combination against Clinical, Drug-Resistant Isolates of E. coli and P. aeruginosa

**Figure 1:**
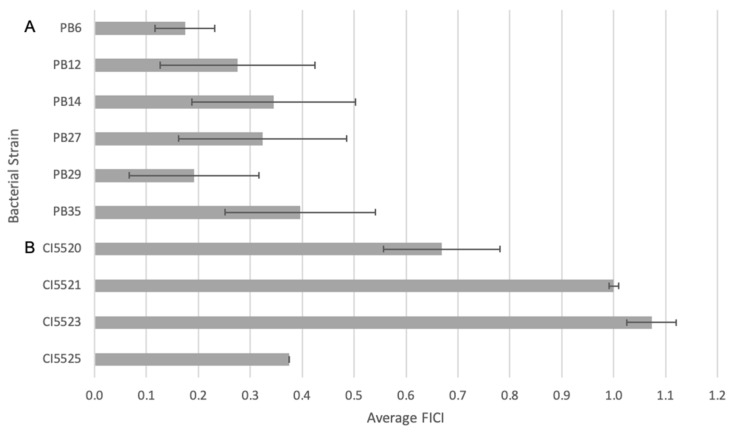
Fractional inhibitory concentration index (FICI) of PMB and LL-37 against (**A**) *E. coli* and (**B**) *P. aeruginosa* CIs, where FICI ≤ 0.5 is synergistic, 0.5 < FICI ≤ 1.0 is additive, 1.0 < FICI ≤ 4.0 is indifferent, and FICI > 4.0 is antagonistic. The synergistic responses of LL-37 and PMB against clinical, drug-resistant isolates of *E. coli* and *P. aeruginosa* were evaluated using checkerboard assays. The microtiter plates were incubated at 37 °C under static conditions for 18–22 h before 100 μL of 0.2 mM resazurin was added to all wells. After an additional incubation of 2 h, the levels of reduced resazurin were quantified at 560 nm. Control wells included MH media and bacteria in the absence of antimicrobial agents as sterility and growth controls, respectively. The mean FICI and standard deviations for 10 different clinical, drug-resistant isolates were calculated from at least three independent experiments.

The initial synergy testing demonstrated synergism between PMB and LL-37 against *E. coli* MG1655 and *P. aeruginosa* PAO1 laboratory strains. To further examine the synergistic effects between the two antimicrobial agents, the FICI was calculated for six pathogenic, extraintestinal *E. coli* clinical isolates (CIs)—PB6, PB12, PB14, PB25, and PB35 [19,20]—as well as for four *P. aeruginosa* CIs—CI5520, 5521, 5523, and 5525 (Figure 1). All isolates showed resistance to two or more antibiotics (Table 1). The combination of LL-37 and PMB led to synergism against all tested *E. coli* CIs. In fact, all the isolates, except for PB35, displayed a stronger synergistic effect compared with the MG1655 laboratory strain. For the studied *P. aeruginosa* CIs, the combination treatment showed synergy against CI5525 (Figure 1), additive synergy to CI5520 and 5521, and indifference to CI5523.

### 2.3. Inhibition of E. coli MG1655 and P. aeruginosa PAO1 Biofilm Formation

Given that the combination of LL-37 and PMB showed synergy against *E. coli* and *P. aeruginosa* planktonic cells (Table 2, Figure 1), we began to investigate its anti-biofilm activity. To examine the inhibition of biofilm growth, various concentrations of PMB were tested as a monotreatment, as well as in combination with 4 μg/mL and 16 μg/mL for *E. coli* and *P. aeruginosa*, respectively. For the concentration of LL-37, ¼ MIC was used as it has been previously shown to reduce bacterial biofilm formation [21]. 

**Figure 2:**
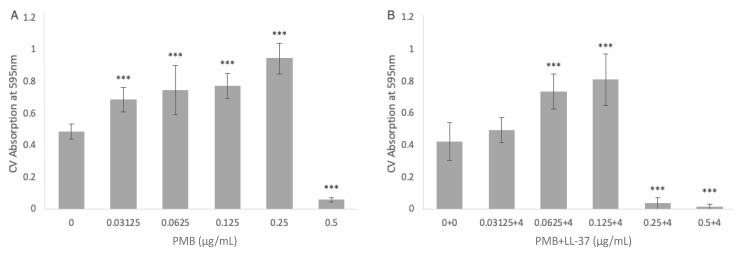
The inhibitory effect of (**A**) PMB alone and (**B**) the combination of PMB + LL-37 on the formation of *E. coli* MG1655 biofilms. The ability of PMB, alone and in combination with LL-37, to inhibit *E. coli* MG1655 biofilm growth was evaluated via crystal violet staining. (**A**) The amount of *E. coli* MG1655 biofilm growth in the presence of single treatments of 0 µg/mL, 0.03125 µg/mL, 0.0625 µg/mL, 0.125 µg/mL, 0.25 µg/mL, and 0.5 µg/mL PMB. (**B**) The amount of *E. coli* MG1655 biofilm growth in the presence of 0 µg/mL PMB + 0 µg/mL LL-37, 0.03125 µg/mL PMB + 4 µg/mL LL-37, 0.0625 µg/mL PMB + 4 µg/mL LL-37, 0.125 µg/mL PMB + 4 µg/mL LL-37, 0.25 µg/mL PMB + 4 µg/mL LL-37, and 0.5 µg/mL PMB+ 4 µg/mL LL-37 treatments. The biofilm biomass was measured at 595 nm after an incubation of 24 h at 37 °C. The mean background absorbance values were subtracted from the results for the test wells. The analysis of variance (ANOVA) tests (***, *p* < 0.001) were calculated for the average values from at least two independent experiments. Each experiment included two independent bacterial cultures, with each condition including data from eight wells of a microtiter plate. Standard deviation bars are shown.

We first investigated *E. coli* MG1655 biofilm formation when treated with 0–0.5 μg/mL PMB monotreatment (Figure 2A). When compared with the no treatment PMB control, 0.03125 μg/mL, 0.0625 μg/mL, 0.125 μg/mL, and 0.25 μg/mL PMB all led to statistically significant increases in *E. coli* biofilm growth (*p* < 0.001, ANOVA). The sole concentration of PMB that yielded a statistically significant reduction in biofilm growth in comparison with the no treatment control was 0.5 μg/mL PMB (*p* < 0.001, ANOVA). When combined with 4 μg/mL LL-37, PMB also led to statistically significant biofilm mass increases at 0.0625 μg/mL and 0.125 μg/mL PMB (*p* < 0.001, ANOVA) (Figure 2B). However, while PMB alone yielded a significant increase in biofilm growth at 0.25 μg/mL, the combination of 0.25 μg/mL PMB + 4 μg/mL LL-37 led to a strong and statistically significant reduction in biofilm growth (*p* < 0.001, ANOVA). 

**Figure 3:**
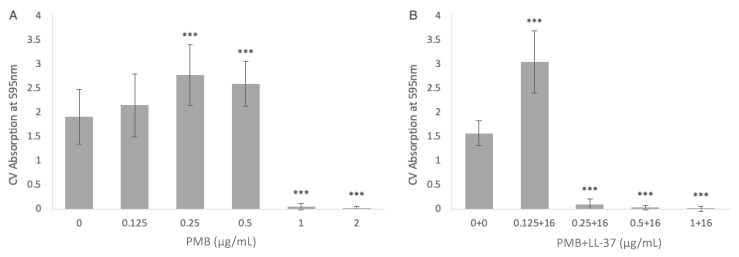
The inhibitory effect of (**A**) PMB alone and (**B**) the combination of PMB + LL-37 on the formation of *P. aeruginosa* PAO1 biofilms. The ability of PMB, alone and in combination with LL-37, to inhibit *P. aeruginosa* PAO1 biofilm growth was evaluated via crystal violet staining. (**A**) The amount of *P. aeruginosa* PAO1 biofilm growth in the presence of single treatments of 0 µg/mL, 0.125 µg/mL, 0.25 µg/mL, and 0.5 µg/mL, 1 µg/mL, and 2 µg/mL PMB. (**B**) The amount of *P. aeruginosa* PAO1 biofilm growth in the presence of 0 µg/mL PMB + 0 µg/mL LL-37, 0.125 µg/mL PMB + 16 µg/mL LL-37, 0.25 µg/mL PMB + 16 µg/mL LL-37, 0.5 µg/mL PMB + 16 µg/mL LL-37, and 1 µg/mL PMB + 16 µg/mL LL-37 treatments. The biofilm biomass was measured at 595 nm after an incubation of 24 h at 37 °C. The mean background absorbance values were subtracted from the results for the test wells. The analysis of variance (ANOVA) tests (***, *p* < 0.001) were calculated for the average values from at least two independent experiments. Each experiment included two independent bacterial cultures, with each condition including data from eight wells of a microtiter plate. Standard deviation bars are shown.

To examine the ability of PMB monotreatment to inhibit *P. aeruginosa* PAO1 biofilm formation, the bacteria were treated with 0–2 μg/mL PMB (Figure 3A). In comparison with the no treatment control, 0.125 μg/mL of PMB did not lead to statistically significant change in biofilm growth. At concentrations of 0.25 μg/mL and 0.5 μg/mL PMB, there was a statistically significant increase in *P. aeruginosa* biofilm growth (*p* < 0.001, ANOVA). Concentrations of 1 μg/mL and 2 μg/mL PMB had significant anti-biofilm activity (*p* < 0.001, ANOVA). When combined with 16 μg/mL LL-37, 0.125 μg/mL PMB led to statistically significant biofilm growth compared with the no treatment control (*p* < 0.001, ANOVA). However, the anti-biofilm activity of *P. aeruginosa* PAO1 occurred at much lower concentrations of PMB when combined with LL-37 (Figure 3B). When used in isolation, 1 μg/mL was needed to cause significant anti-biofilm activity, however in combination with LL-37, anti-biofilm activity was present at 0.25 μg/mL. The inhibitions of biofilm growth at 0.25 μg/mL PMB + 16 μg/mL LL-37, 0.5 μg/mL PMB + 16 μg/mL LL-37, and 1 μg/mL PMB + 16 μg/mL LL-37 were statistically significant compared with the no treatment control (*p* < 0.001, ANOVA). 

### 2.4. Biofilm Eradication of E. coli MG1655 and P. aeruginosa PAO1 Biofilms

The PMB + LL-37 combination exhibited biofilm eradication activity in addition to its biofilm inhibition properties. The combination of 2 μg/mL PMB + 16 μg/mL LL-37 was evaluated using a modified resazurin assay whereby the respiratory activity of pre-grown *P. aeruginosa* PAO1 biofilms was measured over a 6-h period. Due to poor penetration and diffusion of antibiotics in mature biofilm structures, higher doses of antibiotics are needed to treat established biofilms [22]. Therefore, the concentration of PMB was increased to 2 μg/mL from 0.5 µg/mL, which was used in the previous biofilm inhibition experiment. The concentration of LL-37, however, remained the same for the biofilm inhibition and biofilm eradication experiments as AMPs penetrate biofilm structures and can therefore target the embedded bacteria more readily than antibiotics [23]. 

**Figure 4:**
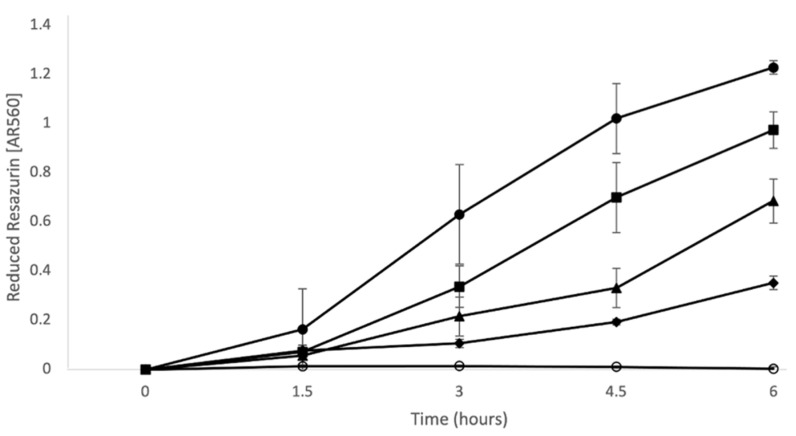
LL-37 and PMB individual versus combined treatment against *P. aeruginosa* PAO1 pre-grown biofilms. *P. aeruginosa* PAO1 biofilms were grown in a 96-well microtiter plate at 37 °C for 24 h. Pre-grown biofilms were treated with 0.1 mM resazurin redox indicator and antimicrobial agents: LL-37 alone (16 µg/mL; closed square), PMB alone (2 μg/mL; closed circle), and PMB + LL-37 combination treatment (2 μg/mL PMB + 16 µg/mL LL-37 closed diamond). Water in the absence of *P. aeruginosa* PAO1 and antimicrobial agents (opened circle) was included as a control for the absorbance of non-reduced resazurin. Control wells containing *P. aeruginosa* PAO1 in the absence of antimicrobial agents were grown in MH media (closed triangle), as the antibiotic and peptide stock solution were prepared in this medium. Every 1.5 h, the amount of reduced resazurin (resorufin) was measured at 560 nm. The timepoints on the image show the mean average and standard deviation of two independent experiments, each performed in triplicate.

The combination of 2 μg/mL PMB + 16 μg/mL LL-37 incurred a decrease in biofilm activity compared with the no treatment conditions, as well as the individual LL-37 and PMB treatments (Figure 4). The respiratory activity of the MH growth control increased at a constant rate until 4.5 h after treatment, where, between 4.5 and 6 h, there was a steeper increase in redox activity. The PMB treatment had the greatest initial increase in redox activity but began to plateau after 4.5 h. The wells containing LL-37 had a more constant increase in respiratory activity that was greater than that of the MH control. When combined at the same concentrations, the PMB + LL-37 combination showed lower AR560 values (corrected absorbance at 560 nm) than all experimental samples, as well as the MH control. The AR560 of the PMB + LL-37 treatment condition had only increased to 0.1 after 3 h, while the no treatment control had nearly double the amount. After 3 h, the bacteria treated with the PMB + LL-37 combination began increasing at a faster rate and rose to 0.35 Au at 6 h of incubation. Despite the increase in activity in the combination wells, the values remained lower than that of the control condition which had an AR560 value of 0.68 Au at 6 h. Biofilms treated with the combination of 2 μg/mL PMB + 16 μg/mL LL-37 displayed statistically significant reductions in AR560 values (*p* < 0.01, Mann–Whitney test) compared with the MH control biofilms. After three hours of observation, the AR560 values of the 2 μg/mL PMB + 16 μg/mL LL-37 treatment were significantly lower than the LL-37 and the PMB individual treatment conditions (*p* < 0.01, Mann–Whitney test). Therefore, treatment of pre-grown PAO1 biofilms with LL-37 and PMB alone triggered significant bacterial growth (*p* < 0.01, Mann–Whitney test), but when combined, bacterial respiratory activity was inhibited. 

The modified resazurin assay was not performed to measure the ability of the PMB + LL-37 combination treatment to eradicate *E. coli* MG1655 biofilms. Due to the high metabolic activity of the bacterium, the indicator could not successfully quantify the respiratory activity of the pre-grown biofilms. The assay relies on the conversion of weakly florescent resazurin to highly fluorescent resorufin. However, the high levels of metabolically active cells triggered a further reaction, forming the non-fluorescent molecule hydroresorufin. The presence of hydroresorufin decreased the absorbance readings, despite the continued increase in biofilm activity [24]. Several methods to slow the biofilm growth proved unsuccessful, including reduction of cell numbers, incubation times, and temperature, as well as modifications to the growth media and washing techniques. In place of the resazurin assay, CV staining was used to measure the biofilm eradication of pre-grown *E. coli* MG1655 biofilms.

**Figure 5:**
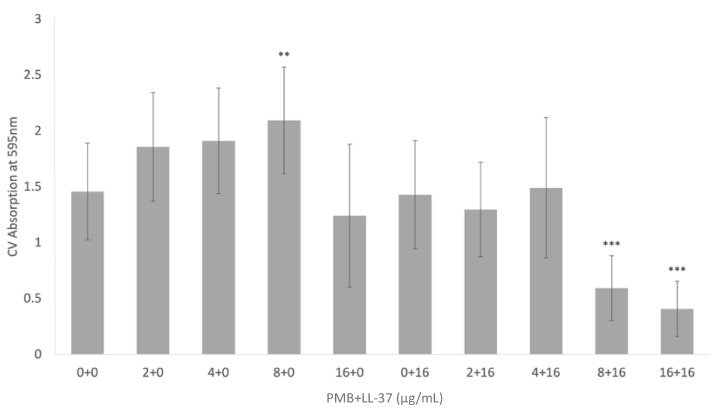
Eradication of *E. coli* MG1655 pre-grown biofilms treated with PMB, LL-37, and the PMB + LL-37 combination treatment. The ability of PMB, alone and in combination with LL-37, to eradicate *E. coli* MG1655 biofilm growth was evaluated via crystal violet staining. *E. coli* MG1655 biofilms were grown in a 96-well microtiter plate at 37 °C for 24 h. Pre-grown biofilms were treated with varying concentrations of PMB (0 µg/mL, 2 µg/mL, 4 µg/mL, 8 µg/mL, and 16 µg/mL), LL-37 (0 µg/mL and 16 µg/mL) and the PMB + LL-37 combination (2 µg/mL PMB + 16 µg/mL LL-37, 4 µg/mL PMB + 16 µg/mL LL-37, 8 µg/mL PMB + 16 µg/mL LL-37, and 16 µg/mL PMB + 16 µg/mL LL-37). Pre-grown biofilms treated with the individual and combined antimicrobial treatments were incubated at 37 °C for 24 h before adding 0.1% crystal violet. The biofilm biomass was measured at 595 nm and the mean background absorbance values were subtracted from the results for the test wells. The analysis of variance (ANOVA) tests (**, *p* < 0.01; ***, *p* < 0.001) were calculated for the average values from at least two independent experiments. Each experiment included independent bacterial cultures, with each condition including data from eight wells of a microtiter plate. Standard deviation bars are shown.

CV staining was used to determine the biomass of pre-grown *E. coli* MG1655 biofilms after treatments with PMB, LL-37, and PMB + LL-37. When pre-grown biofilms were treated with 2 μg/mL and 4 μg/mL PMB, there were insignificant increases to bacterial biomass in comparison with the no treatment control (Figure 5). Treatment with 8 μg/mL PMB, however, led to a statistically significant increase in biomass (*p* < 0.01, ANOVA). Both PMB and LL-37 individual treatment at 16 μg/mL did not result in significant changes relative to the no treatment control. Likewise, when 2 μg/mL and 4 μg/mL PMB were added to 16 μg/mL LL-37, there was no change in biofilm eradication compared with the control. In contrast, adding increasing doses of PMB to 16 μg/mL LL-37 increased biofilm eradication; the combinations of 8 μg/mL PMB + 16 μg/mL LL-37 and 16 μg/mL PMB + 16 μg/mL LL-37 led to statistically significant increases in biofilm eradication when compared with the no treatment control (*p* < 0.001, ANOVA). In addition, the combinations 8 μg/mL PMB + 16 μg/mL LL-37 and 16 μg/mL PMB + 16 μg/mL LL-37 had statistically significant increases in biofilm eradication in comparison with all test conditions (*p* < 0.001, ANOVA). 

**Figure 6:**
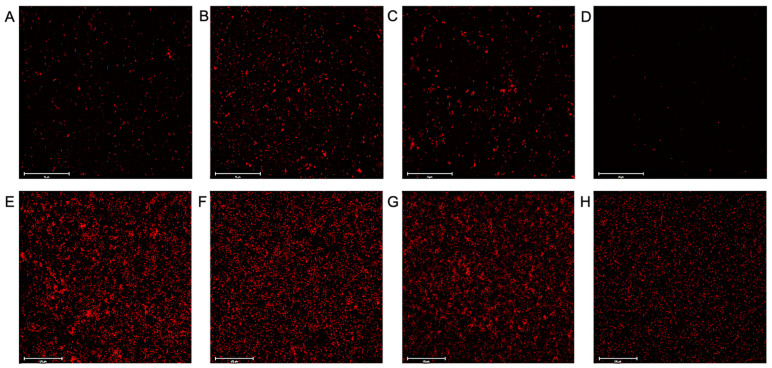
Visualization of LL-37 and PMB individual versus combined treatment of *E. coli* MG1655 and *P. aeruginosa* PAO1 pre-grown biofilms. *E. coli* MG1655 and *P. aeruginosa* PAO1 biofilms were grown in MH in a flat bottom 96-well microtiter plate at 37 °C for 17 h and 24 h, respectively. *E. coli* MG1655 pre-grown biofilms were treated with the following concentrations of antimicrobial agents: (**A**) 0 µg/mL, (**B**) 0.5 µg/mL PMB, (**C**) 8 µg/mL LL-37, or (**D**) 0.5 µg/mL PMB + 8 µg/mL LL-37. *P. aeruginosa* pre-grown biofilms were treated with the following concentrations of antimicrobial agents: (**E**) 0 µg/mL, (**F**) 2 µg/mL PMB, (**G**) 16 µg/mL LL-37, or (**H**) 2 µg/mL PMB + 16 µg/mL LL-37. Treated biofilms were incubated at 37 °C for 2 h (*E. coli* MG1655) and 1 h (*P. aeruginosa* PAO1) prior to the addition of 10 µL of 5 mM CTC stain. The microplates were placed in the 37 °C incubator for 2 h before visualization. Two independent experiments were performed, with each experimental and control condition being repeated in triplicate. Representative images at 40× magnification (*E. coli* MG1655) and 20× magnification (*P. aeruginosa* PAO1) are shown with scale bars indicating 75 µm and 125 µm for *E. coli* MG1655 and *P. aeruginosa* PAO1, respectively.

Fluorescence microscopy with CTC stain was used to visualize the viable cells within the biofilms, and therefore was used to confirm that the PMB + LL-37 combined treatment eradicated pre-grown *E. coli* MG1655 and *P. aeruginosa* PAO1 biofilms. Preliminary testing with *E. coli* MG1655 found that 4 μg/mL LL-37 did not eradicate biofilms, even when combined with increasingly large concentrations of PMB. Therefore, the concentrations of both LL-37 and PMB were increased to account for the increased tolerance of biofilm cells. In comparison with the *E. coli* MG1655 untreated control (Figure 6A), both the 0.5 µg/mL PMB (Figure 6B) and 8 µg/mL LL-37 (Figure 6C) individual treatments stimulated biofilm growth. When compared with the no treatment control, the LL-37 individual treatment led to a 13% increase in biofilm growth, while the PMB treatment yielded the highest bacterial cell coverage with 177% more biofilm growth. In contrast, the 0.5 µg/mL PMB + 8 µg/mL LL-37 combination (Figure 6D) was effective at eradicating pre-grown biofilms, as visualized by a 10-fold reduction in biofilm cells compared with the no treatment control. Additionally, microscopy confirmed the results from the previous biofilm eradication assay, showing that the 2 µg/mL PMB + 16 µg/mL LL-37 treatment caused destruction of pre-grown *P. aeruginosa* PAO1 biofilms. When compared with the *P. aeruginosa* PAO1 untreated control (Figure 6E), the 2 µg/mL PMB (Figure 6F) and 16 µg/mL LL-37 (Figure 6G) individual treatments led to higher biofilm cell numbers, with a 39% and 3% increase, respectively. However, there was a 61% decrease in the amount of biofilm cells in the 2 µg/mL PMB + 16 µg/mL LL-37 treatment (Figure 6H) compared with the no treatment control.

### 2.5. In Vivo Studies Using Galleria mellonella and P. aeruginosa PAO1

**Figure 7:**
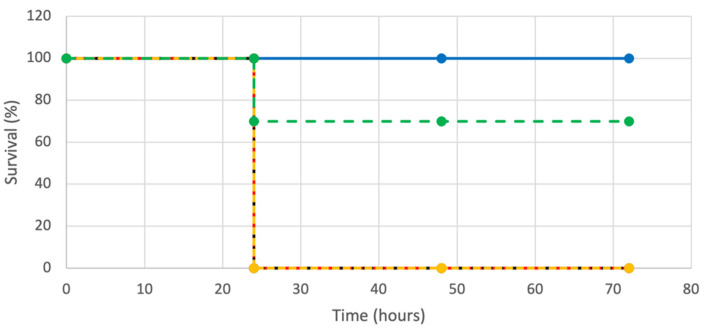
Kaplan–Meier survival curves for *G. mellonella* following injections of *P. aeruginosa* PAO1, with LL-37 and PMB, alone or in combination. One hundred twenty-five *G. mellonella* were injected with 5.0 × 10^1^ *P. aeruginosa* PAO1 cells. After 20 min at 4 °C, the *G. mellonella* were injected with LL-37 and PMB, alone or in combination. Twenty-five *G. mellonella* were injected for each condition with the following antimicrobial agent concentrations: 32 µg/mL LL-37 (black, dotted line), 0.25 µg/mL PMB (yellow, dotted line), and 0.25 µg/mL PMB + 32 µg/mL LL-37 (green, dotted line). Twenty-five *G. mellonella* were included in control conditions of 5.0 × 10^1^ *P. aeruginosa* PAO1 cells in the absence of PMB injection (red, dotted line), and PBS injection with neither bacterial cells nor PMB injections (blue, solid line). The survival of the worms was evaluated every 24 h for 72 h.

The *G. mellonella* wax moth larvae infected with *P. aeruginosa* PAO1 served as an in vivo model to assess the effectiveness of the PMB + LL-37 combination. The *G. mellonella* were injected with either individual or combined treatments of LL-37 and PMB. Preliminary experimentation found that LL-37 alone did not save the *G. mellonella* wax worms from *P. aeruginosa* PAO1 infection, even at increasingly large concentrations. Therefore, the concentration of LL-37 that was injected was 32 µg/mL (½ MIC). Additional preliminary tests were performed with varying concentrations of PMB (10 µg/mL to 0.25 µg/mL) which were injected following PAO1 injection. The lowest concentration of PMB that led to 100% *G. mellonella* death was 0.25 µg/mL and therefore this concentration was chosen for further evaluation. Additional control *G. mellonella* were either injected with a single 10 ul dose of PBS or *P. aeruginosa* PAO1. The PBS no treatment control showed 100% survival, indicating that the physical act of injection was not lethal to the *G. mellonella*. The condition with a PAO1 single dose yielded 0% survival, demonstrating that this concentration of bacterium was lethal to the worms in the absence of antimicrobial treatment. The individual treatments of LL-37 and PMB were not effective in saving the *G. mellonella* from *P. aeruginosa* PAO1 as both conditions resulted in 0% survival at 72 h post-injection (Figure 7). In contrast, the PMB + LL-37 combination treatment had a 70% survival rate 72 h after injecting the worms with the same number of *P. aeruginosa* PAO1 cells. 

### 2.6. Membrane Disruption

**Figure 8:**
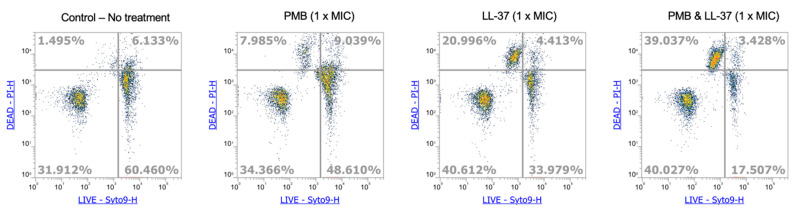
Membrane disruption of *E. coli* MG1655 by PMB and LL-37, alone and in combination. Cells of *E. coli* MG1655 were incubated for 30 min in the presence of MIC concentrations of PMB, LL-37 and PMB + LL-37. Cells were subsequently stained with Syto 9 and propidium iodide for 15 min in the dark. Fluorescence was measured using an ATTUNE NXT Flow Cytometer (Thermo Fisher) until 15,000 events were obtained. Results were analyzed using the Flow Cytometer Software.

It has been previously described that PMB and LL-37 use membrane disruption as a primary mode of action against gram-negative bacteria [15,25]. Additionally, a recent study by Han et al. reports that the membrane permeabilization of LL-37 and PMB work synergistically, such that the MIC of PMB required against *P. aeruginosa* PAO1 and PA103 are lower than for PMB monotreatment [26]. Given the current knowledge, we analyzed the membrane integrity of *E. coli* MG1655 in the presence of PMB, LL-37, as well the PMB + LL-37 combination. After treating the *E. coli* MG1655 with MIC concentrations of PMB, LL-37, and PMB + LL-37, the cells were stained using Syto 9 and propidium iodide (PI) and then analyzed using flow cytometry. In contrast with the membrane-permeable Syto 9, which enters intact cells, the incorporation of PI into the cell is dependent on a permeabilized bacterial membrane and is therefore an indicator for membrane integrity [27,28]. After 45 min of treatment, only ~8% of PMB treated cells and ~21% of LL-37-treated cells were stained with PI (Figure 8). In contrast, 39% of cells treated with the PMB + LL-37 combination were stained with PI, thus exhibiting increased membrane permeabilization compared with the individual treatments. Untreated cells were used as controls, with only 1.5% of cells being stained with PI. These results demonstrate an increase in membrane disruption of the PMB + LL-37 combination-treated cells in comparison with the single antimicrobial treatments.

## 3. Discussion

The increase in bacterial resistance from widespread antibiotic misuse, coupled with the inadequate production of new antimicrobials, has led to antimicrobial resistance becoming one of the top threats to public health [29]. Drug-resistant and multidrug-resistant bacteria are endemic in several locations across the world, with infection rates from these bacteria increasing globally [30,31]. Bacterial resistance has rendered some common infections difficult, or even impossible, to treat [31]. Consequently, resistance is leading to higher medical costs, longer hospital stays, and increased patient mortality [31]. It is crucial that new antimicrobial agents are developed to combat the rise in resistance, and to ensure the continued effective treatment of bacterial infections. One strategy is to combine pre-existing antimicrobial agents that display synergistic effects. Given that the last new antibiotic class used to treat infections was discovered in the late 1980s [32], the modification of known antimicrobial agents is more feasible than the development of new therapeutic agents. In addition, it has been hypothesized that bacteria are less likely to develop resistance when combination treatments are used over individual antimicrobial agents [33]. It is thought that antibiotics alone lack the broad-spectrum mechanism of action and/or pharmacokinetic characteristics that enable rapid access to the bacterial target site [33]. 

A main advantage of synergistic combinations is that it results in lower concentrations of each antimicrobial agent needed for effective antibacterial activities [34]. The ability to use lower concentrations has several benefits, including the lower cost of production, as well as lowering the antimicrobial agents’ toxicity to mammalian cells [34]. Both PMB and LL-37 are known to be toxic to human cells in higher concentrations [35,36]. PMB was approved as a treatment against bacterial infections in the late 1950s however, it was discontinued approximately 20 years later due to its promotion of nephrotoxicity and neurotoxicity in humans [35]. Due to the emergence of multidrug-resistant bacteria, PMB had to be reintroduced in clinical settings as a last-resort antibiotic for gram-negative infections, particularly for carbapenem-resistant *Enterobacteriaceae*, *P. aeruginosa* and *A. baumannii* [35]. Additionally, the toxicity of AMPs, such as LL-37, is one of the major obstacles to developing the peptides as clinically used therapeutic agents [37]. Almaaytah et al., [37], along with other studies [12,38,39] have demonstrated that the synergism between antimicrobial agents reduced the MICs, leading to significantly lower toxicity levels [37]. When combined with several classes of antibiotic, LL-37 displayed strong synergy with PMB against *E. coli* MG1655 and *P. aeruginosa* PAO1 laboratory strains. The PMB + LL-37 combination also showed strong synergy against all tested *E. coli* drug-resistant and CI strains (FICI ≤ 0.5). Although synergy was only observed in ¼ *P. aeruginosa* CIs, the remaining effects were additive (FICI 0.5 to ≤1.0), or indifferent (FICI 1.0 to ≤4.0); no antagonistic responses were observed during the synergy testing. This study provided evidence that the MIC values are lowered when used in combination, however further research is needed to determine whether the combination of PMB + LL-37 does indeed have a lower toxicity profile compared with the individual antimicrobial agents. 

Applications of the PMB + LL-37 combination treatment will likely be topical therapies for bacterial infections. Mahlapuu et al., examined the use of topical LL-37 against hard-to-heal-venous leg ulcers in a prospective randomized placebo-controlled clinical trial [40]. It was concluded that administrations of 0.5 mg/mL and 1.6 mg/mL were safe and well-tolerated in patients [40]. Likewise, a clinical trial examined the use of 0.5 mg/mL LL-37 cream to treat diabetic foot ulcers [41]. The results from this trial have yet to be published [41]. The results from the clinical trial performed by Mahlapuu et al., show that concentration of LL-37 is safe at concentrations that are much higher than the anticipated concentrations used in an LL-37 combination therapy [40]. Chitosan hydrogel + LL-37 was also effective in treating deep tissue injury in a mouse model, without altering the viability of the mouse fibroblast cells [42]. Additionally, PMB is used in combination with neomycin and bacitracin in the Polysporin antibacterial ointment. Polysporin was first patented for use in the United States in 1971, and thus PMB has demonstrated a high safety profile as a topical treatment [43]. 

In the present study, we combined PMB, a peptide antibiotic with high bactericidal activity against gram-negative bacteria but no anti-biofilm properties, with LL-37, an anti-biofilm peptide with moderate antibacterial effects. Features of LL-37, including its small size, net positive charge, large antibacterial spectrum, ability to kill non-metabolically active cells, and immunomodulation, have enabled it to effectively kill biofilms [44]. We have shown that the addition of LL-37 to PMB conferred significant biofilm inhibition and eradication properties. The combination of 0.25 μg/mL PMB + 4 μg/mL LL-37 led to statistically significant prevention of *E. coli* MG1655 biofilm formation whereas 0.25 μg/mL PMB alone did not inhibit biofilms, and in fact resulted in a statistically significant promotion of biofilm growth. Similarly, the combinations of 0.25 μg/mL PMB + 16 μg/mL LL-37 and 0.5 μg/mL PMB + 16 μg/mL LL-37 led to statistically significant inhibitions of *P. aeruginosa* PAO1 biofilm formation while PMB alone, at the same concentrations, led to statistically significant increases in biofilm growth. In terms of biofilm eradication, combinations of 8 μg/mL PMB + 16 μg/mL LL-37 and 16 μg/mL PMB + 16 μg/mL LL-37 had statistically significant reductions in *E. coli* MG1655 biomass compared with all control and individual treatment conditions. Likewise, the 2 μg/mL PMB + 16 μg/mL LL-37 combination had a statistically significant increase in *P. aeruginosa* PAO1 pre-grown biofilm eradication compared with all control and test treatments. The biofilm eradication was confirmed visually with florescent staining, where the combination of 0.5 μg/mL PMB + 8 μg/mL LL-37 led to less viable *E. coli* MG1655 biofilm present compared with the individual antimicrobial treatments. Furthermore, the combination of 2 µg/mL PMB + 16 µg/mL LL-37 demonstrated visible eradication of pre-grown *P. aeruginosa* PAO1 biofilms. 

Synergistic antimicrobial pairs, including PMB + LL-37, represent a promising method to combat biofilm infections as it has been shown that therapeutic concentrations of singular antimicrobial agents are not effective against biofilms [45]. Bjarnsholt et al. have stated that some antibiotics, such as fluoroquinolones, have displayed enhanced anti-biofilm capabilities; yet the complete eradication of biofilms remains a major challenge [46]. It has been proposed that combination therapies may be the only method to achieve total biofilm eradication within an infection [46]. In addition to its anti-biofilm properties, the PMB + LL-37 combination has proven to be effective in an in vivo model. The *G. mellonella* wax worm model has demonstrated that the combination of 0.25 μg/mL PMB + 32 μg/mL LL-37 was synergistic against 5.0 × 10^1^ *P. aeruginosa* PAO1 infection. The PMB + LL-37 combination treatment had 70% survival rate 72 h-post PAO1 injection, whereas there was 0% survival in the LL-37 and PMB individual treatments. A limitation of this study is the minimal number of concentrations of LL-37 and PMB being tested. The concentrations chosen were based on previous literature reports and preliminary testing, however the concentrations have not been optimized. Lower concentrations of antibiotic and/or peptide may yield similar or enhanced antibacterial and/or anti-biofilm effects.

Although the precise mechanism of action of PMB is not clear, it is proposed to disrupt both the outer and inner membranes of bacteria using the ‘self-promoted uptake’ model [35]. One hypothesized mechanism of synergy between LL-37 and PMB is that they target different areas of the bacterial membrane (cytoplasmic versus outer membrane), leading to enhanced membrane perturbations [47]. An alternative mechanism is that one membrane-targeting agent facilitates the entry of an intracellularly acting agent, allowing easier access to its microbial targets [12,48,49,50,51]. LL-37 and PMB both act on the bacterial membrane as their primary target [35,52]; however, LL-37 has intracellular targets as well [53,54,55], including bacterial genes responsible for energy production and metabolism [55]. 

In previous studies, LL-37 has been shown to synergize with antimicrobial agents with several different modes of action, including the inhibition of cell wall synthesis [56], protein biosynthesis [56,57,58,59,60,61], and bacterial DNA synthesis [45,57], as well as membrane disruption [18,45,62]. Synergistic pairs of antibiotics + LL-37 have been reported for both *E. coli* [61,62] and *P. aeruginosa* [18,45,56,57,58,59,60,63]. A recent study by Han et al., described the synergistic effects of PMB + LL-37 against *P. aeruginosa* PAO1 and PA103 planktonic cells. Synergy of the PMB + LL-37 pair was seen through lowered MIC, minimal bactericidal concentration (MBC), FICI, and fractional bactericidal concentration index (FBCI) [26]. Their results suggest that the synergistic effect is due to the shared membrane permeabilization abilities of PMB and LL-37 [26]. The results from our study are consistent with the findings of Han et al., as we found that the PMB + LL-37 contributed to greater *E. coli* MG1655 membrane permeabilization compared with the PMB and LL-37 individual treatment conditions. Additionally, both Morroni et al. [62] and Geitani et al. [18] have found that LL-37 and colistin synergize against multidrug-resistant *E. coli* strains and multidrug-resistant *P. aeruginosa* strains, respectively. Our findings are in accordance with these previous studies as colistin and PMB are both polymyxin antibiotics, and therefore target bacteria via the same mechanisms. In addition, PMB has demonstrated synergy with AMPs other than LL-37 [47,64,65,66,67,68,69,70,71,72]. Although both LL-37 and PMB have shown synergy with different antibiotics/peptides, very few studies have examined their effects against biofilms cells [45,47,56,57,64]. The combination of LL-37 and PMB represents a novel treatment for gram-negative bacterial infections, one that has the potential to be used against drug-resistant and multi-drug resistant bacterial strains. The anti-biofilm properties of the combination make it a promising future treatment given the high resistance of bacteria in the biofilm state and the large prevalence in medical and surgical settings.

## 4. Materials and Methods

### 4.1. Bacterial Strains

Bacterial strains used in this study include the laboratory strains *E. coli* K-12 (MG1655) [73] and *P. aeruginosa* PAO1 [74] as well as the multidrug-resistant, clinical isolates *E. coli* PB6, PB12, PB14, PB27, PB29 and PB35 [19,20] and multidrug-resistant, clinical isolates *P. aeruginosa* CI5520, CI5521, CI5523 and CI5525 [47]. The antibacterial resistance profiles of all *E. coli* and *P. aeruginosa* strains used in this study are presented in Table 1. 

### 4.2. Growth Conditions

All bacterial strains were maintained as stocks at −80 °C. To prepare standard inoculation material, bacterial cells were retrieved from frozen stocks and streaked on Luria Broth (LB)–Lennox agar plates. Plates were incubated overnight at 37 °C and then stored at either 4 °C (for *E. coli* MG1655) or room temperature (for all other strains). Single colonies were subsequently used to inoculate 10 mL of MH broth in a 125 mL flask. Cultures were left to grow overnight at 37 °C and 200 rpm. Prior to all experiments, cells were washed twice with PBS by centrifuging at 10,000 rpm and 20 °C for 5 min. The cells were then diluted in MH broth to an optical density of 0.1 at 600 nm (OD600). Further dilutions may have been performed depending on the experimental protocol. 

### 4.3. Antibiotics and Antimicrobial Peptide

The antibiotics ampicillin, tetracycline, ciprofloxacin, aztreonam, and colistin were purchased from Thermo Fisher Scientific (Waltham, MA, USA). Polymyxin B was purchased from Research Products International (RPI), and gentamicin from VWR Life Science. The human AMP LL-37 was obtained from Peptide Sciences (Henderson, NV, USA). Stock solutions (1 or 2 mg/mL) of all antimicrobials were made by dissolving the powders in sterile distilled water, except for tetracycline which was dissolved in 70% ethanol. Solutions were stored at either 4 °C or −20 °C (according to safety and handling guidelines), and dilutions were prepared on the day of use.

### 4.4. Broth Microdilution Assay

The minimal inhibitory concentrations (MICs) of the six antibiotics and LL-37 with all *E. coli* and *P. aeruginosa* strains were determined using the broth microdilution assay [75]. Two-fold serial dilutions of the antimicrobials were performed in round-bottom 96-well polystyrene microtiter plates with the highest concentration being 128 μg/mL. The prepared bacterial suspensions were then added to the plates, which were incubated under static conditions at 37 °C for 16–24 h. The MICs were determined as the lowest concentration of the antimicrobial that completely inhibited visible cell growth. The final values were calculated based on the mean results of 3–6 independent experiments. 

### 4.5. Checkerboard Assay

The combined antimicrobial action of antibiotic and LL-37 combinations was assessed using the checkerboard technique, as previously described [47]. Briefly, 25 μL of MH broth and 25 μL of LL-37 stock solutions were mixed in the uppermost row of 96-well polystyrene microtiter plates. MH broth was added to the remaining wells and the antibiotic was serially diluted down the rows of the plate. An amount of 50 μL of the antibiotic was diluted in a similar manner across the columns of a separate 96-well plate and then added to the test plate containing the LL-37. The result consisted of a variety of antibiotic-LL-37 concentrations increasing two-folds from left to right and from bottom to top. One column contained no LL-37 and one row contained no antibiotic, designed to evaluate the MIC of each antimicrobial alone. In addition, one column containing no antimicrobial agents served as a positive (growth) control, and another column containing only 100 μL MH broth served as a negative (sterility) control. All wells containing antibiotic and LL-37, alone or in combination, as well as the positive control wells were inoculated with 50 μL of the prepared bacterial suspension. The plates were incubated for 18–22 h at 37 °C under static conditions in a moist environment. To determine the respiratory activity of the bacteria, 100 μL of 0.2 mM resazurin was added to all wells and the plate was left to incubate for another 2 h. The levels of resorufin (reduced resazurin) were evaluated at 560 nm and 620 nm using a BioTek Epoch 2 microplate reader (Santa Clara, CA, USA). The data were subsequently analyzed and the difference between the absorption values at the two wavelengths were calculated. Negative absorption values, along with a lack of color change from blue to pink, indicated absence of bacterial growth. Synergy between the antibiotics and LL-37 was expressed through the fractional inhibitory concentration index (FICI), which is calculated using the following equation:

FICI = (MIC_antibiotic in combination_)/(MIC_antibiotic alone_) + (MIC_LL-37 in combination_)/(MIC_LL-37 alone_)

FICI values were interpreted as synergistic if ≤0.5, additive if between 0.5 and ≤1.0, indifferent if between 1.0 and ≤4.0, or antagonistic if >4.0. At least three independent experiments were performed, from which the mean FICIs and standard deviations were calculated.

### 4.6. Biofilm Biomass Quantification

As PMB was the sole antibiotic to display a synergistic response with LL-37 (i.e., FICI ≤ 0.5), it was chosen for further experimentation. Biofilm biomass quantification was used to assess the prevention of *P. aeruginosa* PAO1 and *E. coli* MG1655 static biofilm formation. In each well of a round-bottom 96-well microtiter plate, 50 μL of MH broth containing either PMB, LL-37, or their combination was inoculated with 50 μL of bacterial suspension at OD600 of 0.001 (10^6^ CFU/mL). The plates were incubated at 37 °C for 18–22 h under static conditions. The medium was aspirated to remove planktonic cells and the wells were washed twice with MH broth. The biofilm formation was quantified using the Crystal Violet (CV) staining method, as described previously [76]. Briefly, 125 μL of 0.1% (*wt*/*vol*) CV solution was added to each test well and the plate was left to stain at room temperature for 10 min. The wells were washed twice with distilled water to remove excess CV and allowed to dry for another 10 min. An amount of 200 μL of 95% (*vol*/*vol*) ethanol was then added to elute the dye. Following an incubation period of 20 min at room temperature, the intensity of CV at 595 nm was quantified using a plate reader. The mean value of the negative control wells with no biofilm growth was subtracted from the values of the test wells. At least three individual experiments were performed. Results from one representative experiment are shown to minimize variations from daily fluctuations in bacterial cell activity. To further eliminate variation, all samples were compared with the no treatment control within the same microtiter plate. To allow for effective data consolidation from different microtiter plates, the biofilm inhibition was represented as a percentage change relative to its no treatment control. For statistical analysis, ANOVA tests were calculated from absorbance readings from up to sixteen wells per condition. 

Biofilm mass quantification was also performed to evaluate the eradication of pre-grown *E. coli* MG1655 biofilm cells. Overnight cultures of *E. coli* MG1655 were diluted to an OD600 of 0.001 (10^6^ CFU/mL). Biofilms were formed by adding 100 μL of *E. coli* MG1655 to 96-well round-bottom microtiter plates, which were incubated at 37 °C for 24 h under static conditions. The wells were washed twice with MH broth before treating the biofilms with the individual and combined treatment of antimicrobial agents. The microtiter plates were incubated for 18–24 h, then washed with 180 μL of 1× PBS. The method for using CV stain to quantify biomass is the same as described for the prevention of *P. aeruginosa* PAO1 and *E. coli* MG1655 static biofilm formation, except that 1× PBS was used to wash the plates instead of distilled water. The mean value of the negative control wells with no biofilm growth was subtracted from the values of the test wells. The analysis of variance (ANOVA) test was calculated for the average values from at least two independent experiments. Each experiment included independent bacterial cultures, with each test condition including data from eight wells of a microtiter plate. 

### 4.7. Resazurin Assay

A modified resazurin assay in 96-well plates was used to measure the effect of LL-37 and PMB on *P. aeruginosa* PAO1. Resazurin is a blue dye which reduces to pink in the presence of metabolically active cells [24]. To perform the assay, *P. aeruginosa* PAO1 overnight cultures were diluted to 10^6^ CFU/mL (OD 0.001) in MH broth. To form biofilms, 100 μL/well of bacteria were inoculated on a 96-well round-bottom microtiter plate and placed in the 37 °C static incubator for 24 h. Each well was washed twice with 150 μL of MH broth. To measure the biofilm activity of *P. aeruginosa* PAO1, PMB (2 μg/mL) and LL-37 (16 μg/mL), alone and in combination, were added to the wells along with 0.1 mM resazurin indicator. All plates contained control samples of bacteria and resazurin in the absence of antimicrobial agents, as well as the resazurin indicator in the absence of both bacteria and antimicrobials. The final volume per well was 150 μL and the plates were incubated at 37 °C under static conditions. An amount of 100 μL samples from each plate were transferred to a new microtiter plate every 1.5 h, and the absorbance at 560 nm and 620 nm was measured using the BioTek Epoch 2 microplate reader. The absorbance reading at 560 nm represented the amount of reduced resazurin (resorufin), while the absorbance at 620 nm measured the residual amount of oxidized resazurin. The corrected A560 (AR560) was calculated using the following formula:

AR560 = A560 − (A620 × (AO560/AO620))

where AO560 and AO620 are the absorbances of the MH broth containing 0.1 mM resazurin in the absence of bacterial cells.

Three individual experiments were performed. Results demonstrate the biofilm activity from one representative experiment to minimize variations from daily fluctuations in bacterial cell activity. Mean values and standard deviations were calculated from the results and statistical analysis was performed using the Mann–Whitney test.

### 4.8. Fluorescence Microscopy

CTC (5-Cyano-2,3-ditolyl tetrazolium chloride) redox dye was used to visualize the capacity in which LL-37 and PMB eradicate pre-formed biofilms. In the oxidized state, CTC is colorless and nonfluorescent; however, it reduces to formazan (CTF), an insoluble, florescent red compound [77]. The electron transport activity of bacteria reduces CTC to CTF, where CTF then accumulates intracellularly in the bacteria [77]. Therefore, CTC staining is an effective method by which to visualize the respiratory activity of bacterial cells [65]. To prepare the pre-grown biofilms, *E. coli* MG1655 overnight cultures were diluted to 10^5^ CFU/mL (OD 0.0001), while *P. aeruginosa* PAO1 overnight cultures were diluted to 10^6^ CFU/mL (OD 0.001). The bacteria were added to a 96-well flat-bottom microtiter plate to a final volume of 100 μL. The *E. coli* MG1655 biofilms were grown at 37 °C for 17 h under static conditions, while the *P. aeruginosa* PAO1 biofilms were grown at 37 °C for 24 h under static conditions. Once the biofilms were formed, the supernatant was removed, and the wells were washed twice with MH broth to remove the planktonic cells. The *E. coli* MG1655 were treated with 100 μL of LL-37 (8 μg/mL) and (0.5 μg/mL) PMB, alone or in combination. The *P. aeruginosa* PAO1 biofilms were treated with 100 μL of LL-37 (16 μg/mL) and (2 μg/mL) PMB, alone or in combination. For both bacteria, an MH control was used with bacteria to visualize the biofilm formation in the absence of antimicrobial agents. Each condition was performed in triplicate. The microtiter plates were put back in the 37 °C static incubator (2 h for *E. coli* MG1655 and 1 h for *P. aeruginosa*) before adding the CTC stain. In each well, 10 μL of CTC stain (5 mM) was added, then the plates were incubated for an additional 2 h in the 37 °C static incubator. Before visualizing using fluorescence microscopy, the supernatant was removed and replaced with 100 μL of fresh MH media to remove background planktonic cells. Representative images from two independent experiments were taken at 20× (*P. aeruginosa* PAO1) and 40× magnification (*E. coli* MG1655). The number of biofilm cells was quantified using Image J 1.53t.

### 4.9. In Vivo Experimentation

In vivo experiments were performed using the *Galleria mellonella* infection model, as described [47]. Briefly, *G. mellonella* larvae were purchased from Gecko Gurl (Lanark, ON, Canada). To assess the virulence of the *P. aeruginosa* PAO1, 25 worms of approximately equal size and weight were assigned to each control or experimental condition. The control groups included the injection of either PBS or PAO1, both in the absence of antibiotic/peptide. In the experimental conditions, the bacteria were first injected into the dorsal side of the larva, and then placed in the 4 °C refrigerator. After 15–30 min, LL-37 (32 μg/mL) and PMB (0.25 μg/mL), both individually and combined, were injected into the worms. The worms were then placed in the 37 °C static incubator, and observed after 24, 48, and 72 h. The *G. mellonella* were observed visually for survival. The main indicator for survival was the presence of movement, while the color of the worms was a secondary indicator, where white worms were healthy and darker worms were either unhealthy or dead. Kaplan–Meier survival curves were used to evaluate the data from three independent experiments. For each experiment, five to ten worms per condition were assessed, with a maximum of five worms being placed in the same petri dish to avoid death due to overcrowding. 

### 4.10. Membrane Disruption

Flow cytometry, with propidium iodide (PI) and SYTO 9 dyes (Live/Dead viability kit, Thermo Fisher), was used to analyze the ability of PMB, LL-37 and the PMB + LL-37 combination to disrupt *E. coli* MG1655 cell membranes. Methods were as described by Yasir et al., with some modifications [27]. Briefly, cells of *E. coli* MG1655 were grown on agar plates for 18 h at 37 °C and subsequently transferred with an inoculation loop into 1 mL of Hepes 5 mM pH 7.2 with 150 mM NaCl and 20 mM glucose. Cell suspension samples were adjusted to a cell number of 1 × 10^6^ CFU/mL. PMB, LL-37, or the PMB + LL-37 combination was added at MIC concentrations and the samples were incubated for 30 min at room temperature. Cells were stained with PI and SYTO 9 according to the manufactures protocols and incubated for an additional 15 min at room temperature in the dark. Fluorescence was then measured using an ATTUNE NXT Flow Cytometer (Thermo Fisher Scientific, Waltham, MA USA) until 15,000 events were obtained. Results were analyzed using the Attune Cytometric Software (Thermo Fisher Scientific, Waltham, MA, USA).

## 5. Conclusions

The results from the present study demonstrate that LL-37 synergizes with PMB against *E. coli* and *P. aeruginosa* cells. The synergistic response was seen against planktonic cells of laboratory strains, as well as drug-resistant and CI strains. Importantly, the PMB + LL-37 combination treatment displayed biofilm inhibition and eradication properties that were superior to the individual treatment conditions. Combinations with antibiotics may be an effective method to improve LL-37 as an antibacterial and anti-biofilm agent and may solve several issues preventing it from gaining regulatory approval, including its high cost, toxicity to human cells, and susceptibility to degradation. Likewise, combinations with PMB may improve its toxicity profile, reducing its side effects for patients, and potentially making it safe for use beyond last-resort gram negative infections. This study provides a strong basis for further research on the PMB + LL-37 combination and suggests that other AMP-antibiotic pairs should be investigated for use against drug-resistant and multi-drug resistant bacteria. 

## Figures and Tables

**Table 1:** Resistance profile of *E. coli* and *P. aeruginosa* strains used in this study.

	MIC (µg/mL)						
Bacterial Strain	Ampicillin	Tetracycline	Ciprofloxacin	Gentamicin	Aztreonam	Polymyxin B	LL-37
*E. coli*							
MG1655	8	2	0.0156	1	0.25	0.06	16
PB6	>256	64	0.03	2	0.13	0.13	32
PB12	>256	>64	>32	4	0.25	0.25	32
PB14	>256	>64	>64	1	0.13	0.25	64
PB27	>256	>64	0.02	>64	1	0.13	64
PB29	>256	>64	0.0625	128	64	0.13	64
PB35	>256	>64	0.06	>64	64	0.25	64
*P. aeruginosa*							
PAO1	>64	8	0.06	0.5	4	0.125	64
CI5520	>64	32	1	2	8	0.25	64
CI5521	>64	16	1	64	4	0.25	64
CI5523	>64	32	0.5	4	8	0.25	64
CI5525	>64	8	4	64	0.5	0.25	32

**Table 2:** Fractional inhibitory concentration index (FICI) for antibiotics paired with LL-37 against *E. coli* and *P. aeruginosa* lab strains.

	FICI					
Bacterial Strain	Ampicillin	Tetracycline	Ciprofloxacin	Gentamicin	Aztreonam	Polymyxin B
*E. coli* MG1655	1	1	0.5	1	0.75	0.37
*P. aeruginosa* PAO1	n.d.	0.56	0.56	1.5	2	0.31

FICI ≤ 0.5 is synergistic, 0.5 < FICI ≤ 1.0 is additive, 1.0 < FICI ≤ 4.0 is indifferent, and FICI > 4.0 is antagonistic. n.d.: not determined.

## Data Availability

Not applicable.
